# Optimal Combinations of Chemotherapy and Radiotherapy in Low-Grade Gliomas: A Mathematical Approach

**DOI:** 10.3390/jpm11101036

**Published:** 2021-10-16

**Authors:** Luis E. Ayala-Hernández, Armando Gallegos, Philippe Schucht, Michael Murek, Luis Pérez-Romasanta, Juan Belmonte-Beitia, Víctor M. Pérez-García

**Affiliations:** 1 Departamento de Ciencias Exactas y Tecnología Centro Universitario de los Lagos, Universidad de Guadalajara, Enrique Díaz de León 1144, Colonia Paseos de la Montaña, Lagos de Moreno C.P. 47460, Jalisco, Mexico; armando.gallegos@academicos.udg.mx; 2 Universitätsklinik für Neurochirurgie, Bern University Hospital, CH-3010 Bern, Switzerland; Philippe.Schucht@insel.ch (P.S.); Michael.Murek@insel.ch (M.M.); 3 Radiation Oncology Service, Salamanca University Hospital, 37007 Salamanca, Spain; lapromasanta@saludcastillayleon.es; 4 Department of Mathematics, Mathematical Oncology Laboratory (MOLAB), Universidad de Castilla-La Mancha, Avda. Camilo José Cela, 3, 13071 Ciudad Real, Spain; juan.belmonte@uclm.es (J.B.-B.); victor.perezgarcia@uclm.es (V.M.P.-G.)

**Keywords:** mathematical oncology, temozolomide, radiation therapy, mathematical modeling, low-grade gliomas

## Abstract

Low-grade gliomas (LGGs) are brain tumors characterized by their slow growth and infiltrative nature. Treatment options for these tumors are surgery, radiation therapy and chemotherapy. The optimal use of radiation therapy and chemotherapy is still under study. In this paper, we construct a mathematical model of LGG response to combinations of chemotherapy, specifically to the alkylating agent temozolomide and radiation therapy. Patient-specific parameters were obtained from longitudinal imaging data of the response of real LGG patients. Computer simulations showed that concurrent cycles of radiation therapy and temozolomide could provide the best therapeutic efficacy in-silico for the patients included in the study. The patient cohort was extended computationally to a set of 3000 virtual patients. This virtual cohort was subject to an in-silico trial in which matching the doses of radiotherapy to those of temozolomide in the first five days of each cycle improved overall survival over concomitant radio-chemotherapy according to RTOG 0424. Thus, the proposed treatment schedule could be investigated in a clinical setting to improve combination treatments in LGGs with substantial survival benefits.

## 1. Introduction

Adult supratentorial WHO grade II diffuse low-grade gliomas (LGGs) are slowgrowing primary brain tumors that are, in general, incurable due to their infiltrative nature. However, active surgical and oncological treatment may lead to patient survival exceeding 10 years following diagnosis [1].

Treatment typically consists of surgery followed by observation, radiotherapy, chemotherapy, or chemoradiation [2]. The decision as to the specific combination of therapies to be used on each patient is based on the qualitative consideration of different variables including age, tumor grade, performance status, tumor histology and location [3].

Systemic treatments play an important role in the management of LGGs. Procarbazine, lomustine, and vincristine (PCV) and temozolomide (TMZ) have been shown to be effective against low grade gliomas [4,5,6]. Today, both therapies are widely recommended by recent clinical practice guidelines [7,8]. Due to its favorable toxicity profile, prolonged TMZ treatment is a relevant option either as upfront or as adjuvant treatment [9].

With regard to combination treatments, Radiation Therapy Oncology Group (RTOG) 9802, a prospective clinical trial, showed improvements in both progression-free survival (PFS) and overall survival (OS) with six cycles of adjuvant procarbazine, lomustine, and vincristine (PCV) chemotherapy following radiation, when compared with RT alone [10].

There is evidence that the combination of chemotherapy and radiotherapy could be a beneficial strategy for the treatment of LGGs [10]. However, large-scale prospective studies are lacking concerning the combination of radiotherapy with TMZ. In high-grade gliomas (HGG), TMZ used as concurrent and adjuvant chemotherapy has become the standard of care [11]. However, in LGGs, further studies are necessary to ascertain the role of TMZ when used in addition to RT. RTOG 0424, a single-arm phase II study, combined concurrent and adjuvant TMZ with RT to treat patients with high-risk LGG [12]. The 3-year OS rate was 73.1%, which exceeded historical controls. Randomized clinical trials are under way to confirm the advantage of TMZ chemotherapy added to RT for patients with high-risk LGGs [13].

Thus, it seems that the combination of chemotherapy and radiotherapy improves PFS as against chemotherapy or radiotherapy alone for LGGs. The question of treatment schedule then arises: Is a sequential or concurrent treatment the best post-surgery therapeutic option in high-risk gliomas? Clinical trials studying combination treatments of RT and TMZ have adopted the intensive treatment pattern found to be useful in HGGs, consisting of concurrent and adjuvant chemotherapy [11]. However, the natural history of the disease is very different for HGGs and LGGs.

Because of the high cost, human effort, and the time and ethical issues involved in clinical trials and basic medical research, mathematical modeling and analysis can potentially contribute to the discovery of better cancer treatment delivery regimens. This is specially true for combination cancer therapies. Due to the many possible combinations, obtaining an optimal toxicity–efficacy balance is an increasingly complex task. Consequently, standard empirical approaches to optimizing drug dosing and scheduling in patients are now of limited utility. Mathematical modeling can substantially advance this practice through improved rationalization of therapeutic strategies [14,15].

A number of mathematical studies have constructed in-silico models to determine the optimal delivery scheme for either radiation therapy [16,17,18,19,20,21] or chemotherapy [22,23,24,25,26,27] alone. However, although mathematical models have potential for the study of optimal combination treatments [28], no studies have addressed computationally the question of what might be the best combination scheme of TMZ and RT for LGGs.

In this paper, we construct a mathematical model of LGG response to TMZ and RT, describing the longitudinal tumor volumetric dynamics using patient data. We find the parameter regimes best describing the response to treatments. We then use the mathematical model to carry out “in-silico” experiments to explore different treatment regimes. Our results show that concurrent cycles of TMZ and RT could provide substantial survival improvements over concomitant radio-chemotherapy according to RTOG 0424 [12].

## 2. Materials and Methods

### 2.1. Patient Population

Our initial patient dataset consisted of 84 biopsy/surgery confirmed LGG patients, followed by magnetic resonance imaging (MRI) at Bern University Hospital between 1990 and 2013. From that dataset, we chose patients satisfying the following inclusion criteria: (i) tumors treated with TMZ and/or RT; (ii) availability of at least 1 MRI before the start of treatment; (iii) availability of at least one MRI after the end of the treatment; (iv) no treatment other than RT and/or TMZ given in the period of study; (v) no malignant transformation in the period of study; (vi) Ki-67 labeling index ≤6% when available. A total of 17 patients satisfied the inclusion criteria and were classified according to the 2016 WHO criteria as having either astrocytoma or oligodendroglioma (Table 1).

As outlined in Table 1, nine of the patients considered were classified as having oligodendroglioma, six as having astrocytoma, one with oligoastrocytoma, and one as histology not available. Treatment received was TMZ for eight of these, RT for seven, concurrent RT and TMZ for one and sequential RT and TMZ for another one. Patient ages ranged from 28 to 58 years and the Ki-67 labeling index on diagnosis ranged from 1% to 6%.

### 2.2. Image Acquisition and Radiological Measurements of Tumor Size

Radiological glioma growth was quantified by measurements of tumor diameters on successive MRIs (T2/FLAIR sequences). We computed the tumor volumes using the ellipsoidal approximation. The three largest tumor diameters ($D_{1}$, $D_{2}$, $D_{3}$) were measured according to three orthogonal reference planes (axial, coronal and sagittal), and the tumor volumes were estimated using, as stated, the ellipsoidal approximation: $V=(D_{1}\;\cdot \;D_{2}\;\cdot \;D_{3})/2$, following standard clinical practice [29,30]. To estimate the error of the methodology, we took a different set of glioma patients from another study [31] and compared their volumes, as computed accurately using a semi-automatic segmentation approach, with those computed using the ellipsoidal approximation. Mean differences were 18%, which was the reference level taken for the error in the volume computations.

### 2.3. Mathematical Model

To provide a mathematical description of the growth of LGGs and how they respond to RT and TMZ, we assumed that in the absence of treatment the tumor consists of proliferative P(t) and quiescent Q(t) cells. Before the malignant transformation of the tumor, it will keep a low genetic heterogeneity, proliferative cells would grow exponentially, and there would be a bidirectional flow from Q(t) to P(t), accounting for the cells’ ability to switch from a dormant state to the cell cycle and back [32]. These assumptions are not expected to hold for high-grade gliomas, which are known to have a high level of genetic/phenotype heterogeneity and thus different compartments corresponding to subpopulations with biological behavior should be incorporated to the model. We assumed that no additional mutations appear in LGGs for long periods of time, leading to genotypes with different biological behaviors. When TMZ or RT are administered, they damage only proliferative cells [33,34]. TMZ produces early death, accounting for necrosis, autophagy and druginduced apoptosis, with rate $\alpha_{1}$, and delayed death through mitotic catastrophe, with rate $\alpha_{2}$[26], the latter resulting in a population of lethally damaged cells D(t) that die with rate $\beta_{3}$. RT at the doses used for LGG treatment (1.8–2.2 Gy) kills cells mostly through mitotic catastrophe. Thus, it was assumed that each radiation dose *d* in the RT course would move a fraction $1-S_{p}\left(d\right)$ of P(t) cells into the compartment of damaged cells D(t).

Recent work has considered the alternative of adding a delayed death term in the model equations for tumor cells, rather than including the compartment D(t) explicitly in the model [35]. We expect that approach, which does not reduce the number of free parameters, to be equivalent to the one used in this paper.

There is a key biological process that has been omitted in previous mathematical models describing LGG response to RT: the fact that RT stimulates proliferation of quiescent cells [36,37,38]. To account for this process, we will consider that a fraction $1-S_{q}\left(d\right)$ of quiescent cells are stimulated to re-enter the cell cycle for each radiation dose *d*, thus being transferred into the compartment of proliferative cells P(t). Studies regarding repopulation during chemotherapy are scarce, and the mechanisms behind the process are still not clearly understood [39,40], but it seems that there is no similar process in the action of TMZ, or at least that it is not quantitatively as relevant as with RT.

Finally, C(t) describes the concentration of TMZ in brain and tumor tissue. In this paper, we will assume a simple pharmacokinetics and consider that TMZ is cleared at a constant rate λ, leading to an exponential decay of the concentration.

**Figure 1:**
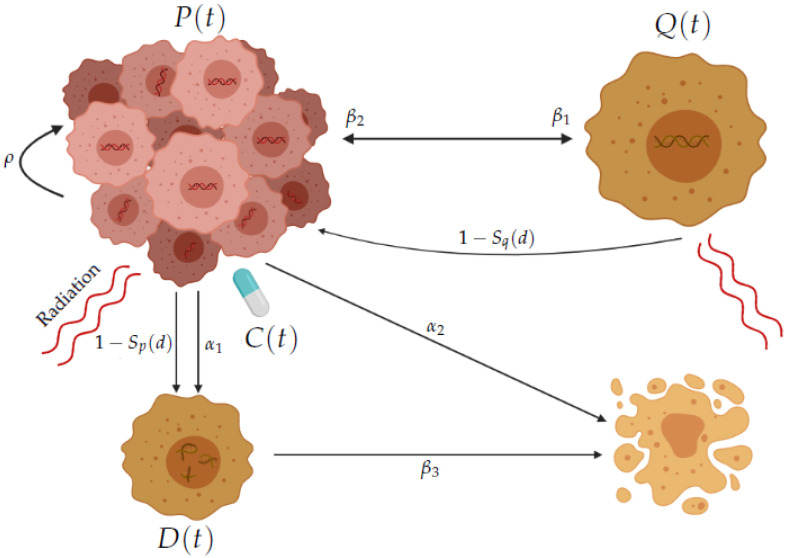
Diagram illustrating the model interactions. Proliferative cells P(t) grow at rate ρ, some enter a quiescent state Q(t) at rate $\beta_{1}$ and vice versa at rate $\beta_{2}$. Radiation damages a fraction $1-S_{p}\left(d\right)$ of P(t) cells to produce a subpopulation of lethally damaged cells D(t) that die at rate $\beta_{3}$, and also stimulates a fraction $1-S_{q}\left(d\right)$ of Q(t) to proliferate. Chemotherapy C(t) causes some P(t) cells to undergo delayed death (as radiation does) at rate $\alpha_{1}$ and immediate death at rate $\alpha_{2}$.

Figure 1 displays a diagram of the biological processes accounted for in our mathematical model.

The equations of the mathematical model are given by the following system of ordinary differential equations:

(1a) $$\begin{array}{cccc} \; & \frac{dP}{dt} & = & \rho P-\beta_{1}P+\beta_{2}Q-\alpha_{1}PC-\alpha_{2}PC, \end{array}$$

(1b) $$\begin{array}{cccc} \; & \frac{dQ}{dt} & = & \beta_{1}P-\beta_{2}Q, \end{array}$$

(1c) $$\begin{array}{cccc} \; & \frac{dD}{dt} & = & \alpha_{1}PC-\beta_{3}D, \end{array}$$

(1d) $$\begin{array}{ccc} \frac{dC}{dt} & = & -\lambda C. \end{array}$$

We assumed the number of cells to be proportional to the volumes occupied by each tumor subpopulation, and thus P(t), Q(t) and D(t) were measured in cm${}^{3}$. In this way, total tumor mass P+Q+D was considered to be proportional to total tumor volume. Since the Ki-67 labeling index provides a measure of the fraction of cells proliferating in tumor tissue, we assumed it to be given by the dimensionless ratio P/(P+Q).

For the particular (but important) case for which there is no treatment, that is, C(t)≡0, we obtain the system:

(2a) $$\begin{array}{cccc} \; & \frac{dP}{dt} & = & \rho P-\beta_{1}P+\beta_{2}Q, \end{array}$$

(2b) $$\begin{array}{ccc} \frac{dQ}{dt} & = & \beta_{1}P-\beta_{2}Q. \end{array}$$

Exact solutions for this system of linear differential equations can be found in the Supplementary Information (SI) Section S1.

The initial time, corresponding to the first MRI study, was denoted by $t_{0}$. Initial conditions for Equation (1) were taken to be $P_{0}=P\left(t_{0}\right)$, $Q_{0}=Q\left(t_{0}\right)$, $D\left(t_{0}\right)=C\left(t_{0}\right)=0$. Let $f(t_{j}^{-})=\;$lim${}_{t\to t_{j}^{-}}f\left(t\right)$ and let $C_{*}$ be the fraction of each chemotherapy dose reaching brain tumor tissue. Drug administration was described as impulses for the times $t_{j}$, so that

(3a) $$\begin{array}{ccc} P(t_{j}) & = & P(t_{j}^{-}), \end{array}$$

(3b) $$\begin{array}{ccc} Q(t_{j}) & = & Q(t_{j}^{-}), \end{array}$$

(3c) $$\begin{array}{ccc} D(t_{j}) & = & D(t_{j}^{-}), \end{array}$$

(3d) $$\begin{array}{cccc} \; & C(t_{j}) & = & C\left(t_{j}^{-}\right)+C_{*}. \end{array}$$

Radiation therapy fractions was simulated as impulses as in previous studies [17,19]. We assumed *n* radiation doses *d* given at times $t_{1},t_{2},\cdots ,t_{n}$, so that after each irradiation event at time $t_{k}$ we get

(4a) $$\begin{array}{cccc} \; & P(t_{k}) & = & S_{p}\left(d\right)P\left(t_{k}^{-}\right)+\left[1-S_{q}\left(d\right)\right]Q\left(t_{k}^{-}\right), \end{array}$$

(4b) $$\begin{array}{cccc} \; & Q(t_{k}) & = & S_{q}\left(d\right)Q\left(t_{k}^{-}\right), \end{array}$$

(4c) $$\begin{array}{cccc} \; & D(t_{k}) & = & D\left(t_{k}^{-}\right)+\left[1-S_{p}\left(d\right)\right]P\left(t_{k}^{-}\right), \end{array}$$

(4d) $$\begin{array}{ccc} C(t_{k}) & = & C(t_{k}^{-}). \end{array}$$

When both therapies are administered simultaneously at times $t_{k}$, Equation (4) become

(5a) $$\begin{array}{cccc} \; & P(t_{k}) & = & S_{p}\left(d\right)P\left(t_{k}^{-}\right)+\left[1-S_{q}\left(d\right)\right]Q\left(t_{k}^{-}\right), \end{array}$$

(5b) $$\begin{array}{cccc} \; & Q(t_{k}) & = & S_{q}\left(d\right)Q\left(t_{k}^{-}\right), \end{array}$$

(5c) $$\begin{array}{cccc} \; & D(t_{k}) & = & D\left(t_{k}^{-}\right)+\left[1-S_{p}\left(d\right)\right]P\left(t_{k}^{-}\right), \end{array}$$

(5d) $$\begin{array}{ccc} C(t_{k}) & = & C\left(t_{k}^{-}\right)+C_{*}. \end{array}$$

The cell–kill parameter $S_{p}$ accounts for all radiation damage that cannot be repaired and will eventually lead to cell death in a delayed form through the population D(t).

### 2.4. Parameter Estimation

#### 2.4.1. TMZ Concentration in the Brain

The usual chemotherapy schedule consists of cycles of 28 days, with five TMZ oral doses on days 1 to 5 and a break on days 6 to 28. The standard dose per day is c= 150 mg per m${}^{2}$ of patient body surface, which is usually around 1.6 m${}^{2}$ for women and 1.9 m${}^{2}$ for men [41]. To further allow for drug loss during transport to the brain we calculated the value of the dose reaching the tumor as

(6) $$C_{*}=\gamma \;\cdot \;c\;\cdot \;b,$$

where γ is the fraction of TMZ getting to 1 cm${}^{3}$ of brain interstitial fluid (from a unit dose) and *b* is the patient’s body surface. γ can be calculated using the value of maximal TMZ concentration $C_{max}$ for a dose of 150 mg/m${}^{2}$ taken from the literature [42]. The time to reach peak drug concentration in the brain is less than two hours and thus negligible in comparison with the other time scales in the model. We chose to set the drug concentration reached by each standard dose *c* to the value $C_{max}=0.6\;\mu$g/cm${}^{3}$ as in Ref. [25].

The dose of TMZ given according to RTOG 0424 [12] during the concurrent stage is 75 mg/m${}^{2}$/day [11]. Thus, in that treatment stage we set the drug concentration in brain tumor tissue equal to $C_{max}/2$.

Using the value of TMZ half-life clearance time $t_{1/2}$ [43], and following the same methodology as in Ref. [25] we determined the rate of drug decay

(7) $$\lambda =-\frac{\ln(1/2)}{t_{1/2}}.$$

A summary of the parameter values involved in the calculation of the TMZ pharmacokinetics is presented in Table 2.

#### 2.4.2. Parameter Fitting

Following the conventional RT administration schedule, consisting of 30 fractions of 1.8 Gy given from Monday to Friday for six consecutive weeks [21], we fixed the radiation dose d=1.8 Gy. The parameters ρ, $\beta_{1}$, $\beta_{2}$, $\beta_{3}$, $\alpha_{1}$, $\alpha_{2}$, $S_{p}$(1.8 Gy) and $S_{q}$(1.8 Gy) and the initial populations $P_{0}$ and $Q_{0}$ were fitted for each patient’s volumetric data using the Matlab (R2020b, The MathWorks, Inc., Natick, MA, USA) function fmincon.

Parameter fitting was performed for each patient by minimizing the quadratic error between longitudinal volumetric data $\left(t_{j},v_{j}\right)$ and simulation results $\left(t_{j},V\left(t_{j}\right)\right)$ obtained from Equations (1) and either (3), (4) or (5), that is,

(8a) $$e\left(\rho ,\beta_{1},\beta_{2},\beta_{3},\alpha_{1},\alpha_{2},S_{p},S_{q}\right)=\sqrt{\sum_{i=1}^{r}\frac{{\left(v_{j}-V\left(t_{j}\right)\right)}^{2}}{r}}.$$

At surgery time *T*, at which the Ki-67% is obtained, we imposed the condition:

(8b) $$\frac{100P(T)}{P(T)+Q(T)}-\mathrm{Ki}67\%=0.$$

Finally, for the initial volume, we required that:

(8c) $$P_{0}+Q_{0}\in \left[0.82v_{0},1.18v_{0}\right]\;{\mathrm{cm}}^{3}.$$

Equation (8) were used to obtain the parameter values. Table 3 displays the results obtained for each patient in our dataset. Time between RT and TMZ for patient 151 was very long (11 years), and distinct values of the Ki-67 labeling index were obtained at the two time points. Thus, different parameter sets were obtained for each treatment.

The numerical solutions of Equation (1) together with (3), (4) or (5) were computed using the Matlab function ode45.

### 2.5. RT-Only Virtual Experiments

We performed simulations for the patients who received only RT, which consisted of shifting the RT starting time to later times to test if the model results were in agreement with the observations in the clinical trial by Van den Bent et al. [44], who found that deferring RT in LGGs did not have an effect on survival (see Section 3.1.2).

We also simulated the tumor behavior when the time interval between RT sessions was increased. To take into account the 18% error in real tumor volumes, we choose randomized tumor volumes within the range defined by error and we computed the value of the parameters best describing the tumor evolution (see Section 3.2).

### 2.6. In-Silico Patients

The data available in Table 3 provide either parameters for RT or TMZ, but only one patient received both treatments concurrently. Then, in order to construct virtual patients through whom to compare the efficacy of combination treatments, we computed the most representative ranges for each parameter in Table 3, that is, $P_{0}+Q_{0}\in \left[2.52,146\right]$ cm${}^{3}$, Ki-67${}_{0}$∈{1,1.5,⋯,6}%, ρ∈[0.01,0.15] 1/day, $\beta_{1}\in \left[0.23,10.60\right]$ 1/day, $\beta_{3}\in \left[1\times 10^{-5},0.021\right]$ 1/day, $S_{q}\in \left[0,0.94\right]$, $S_{p}\in \left[0,0.7\right]$, $\alpha_{1}\in \left[2.3,45\right]$ cm${}^{3}$/(μg day) and $\alpha_{2}\in \left[0.37,20\right]$ cm${}^{3}$/(μg day).

The parameters for adding a virtual therapy or creating an in-silico patient were then chosen by randomly sampling the above corresponding intervals with uniform distribution, and $\beta_{2}$ was calculated using

(9) $$\beta_{2}={\mathrm{Ki}-67}_{0}\%\left(\frac{\beta_{1}}{100-{\mathrm{Ki}-67}_{0}\%}-\frac{\rho }{100}\right).$$

This equation was obtained by taking the limit P(t)/(P(t)+Q(t)) as *t* goes to infinity in the analytic solution of Equation (1) (see SI Section S1). In this way, we ensure values of Ki-67 in the range characteristic of low-grade gliomas.

For radiation therapy treatment, the number of fractions was taken to be 30, which is in line with usual clinical practice. For chemotherapy treatments, we assumed the number of TMZ cycles to be 12, that is, the average number of cycles received by the real patients included in this study (see Table 3).

### 2.7. In-Silico Trial Comparing Different Treatment Modalities/Schedules

To find the best therapeutic approach for large patient cohorts, we designed a threearm in-silico trial. A total of 3000 virtual patients were created by choosing parameters as described in Section 2.6.

Patients in each arm were randomized for the following treatments:

I. Control group without treatment;
II. RT (1.8 Gy)+TMZ (concurrently 75 mg/m${}^{2}$/day and post-radiation 150–200 mg/m${}^{2}$/ day) according to RTOG 0424. For simplicity, we assumed a fixed-dose equal to 150 mg/m${}^{2}$/day in all TMZ cycles;
III. RT+TMZ matching the doses of RT (1.8 Gy) to those of TMZ (150 mg/m${}^{2}$/day) in the first five days of each cycle (of length 28 days).

Using the MatSurv MATLAB package [45], Kaplan–Meier survival curves for overall survival were then calculated. A death event was assumed to happen when tumor volume reached a fatal size (V${}_{F}$) randomly distributed between 113.1 and 268.08 cm${}^{3}$, which is satisfied when V${}_{F}-\left(P_{0}+Q_{0}\right)>40$ cm${}^{3}$, to avoid $P_{0}+Q_{0}\geq$ V${}_{F}$. In line with other work, Pérez-García et al. [26], where a fatal volume for LGGs was assumed to be 268.08 cm${}^{3}$.

As low-grade gliomas are less aggressive and their size is measured in T2/FLAIR MRI sequences, we assumed a slightly larger volume and added the variability as a surrogate of tumor location, which substantially influences the critical tumor size. Patients who only reached the fatal volume after 15 years were considered censored events.

## 3. Results

### 3.1. Validation of the Mathematical Model

#### 3.1.1. The Mathematical Model Given by Equation (1) Described the Tumor Longitudinal Dynamics in Patients

The mathematical model was fitted for each patient as described in Section 2.4, and best parameters obtained as listed in Table 3. The model was flexible enough to describe the dynamics in all patients faithfully.

**Figure 2:**
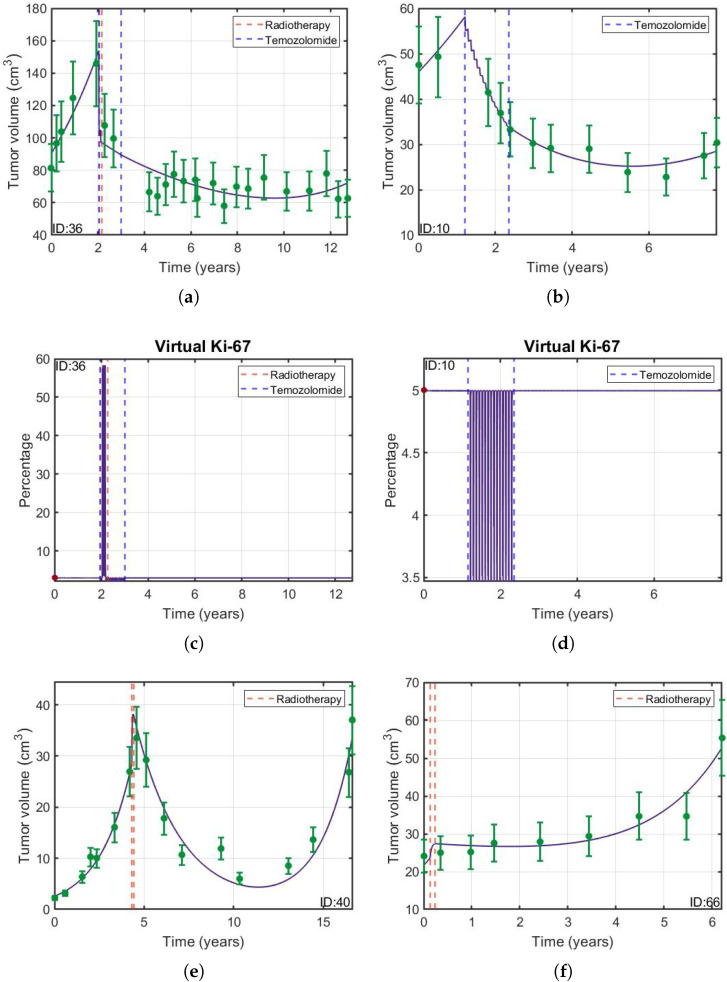

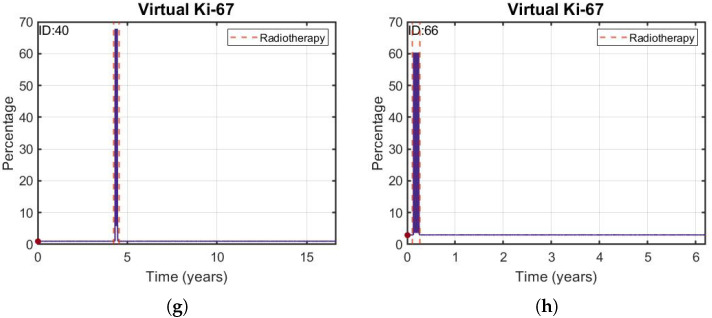
(**a**,**b**,**e**,**f**) Examples of longitudinal volumetric dynamics (green circles) with their respective 18 % error bars (green vertical lines) and best fits obtained using Equation (1) (solid purple line). Data shown correspond to patients 10,36,40 and 66 (green circles). (**c**,**d**,**g**,**h**) Time evolution of 100P(t)/(P(t)+Q(t)) interpreted as virtual (solid purple line) and initial (red circle) Ki-67 labeling index. The vertical dashed lines indicate the onset and end times of the treatments received by each patient (either RT, QT or both).

**Figure 3:**
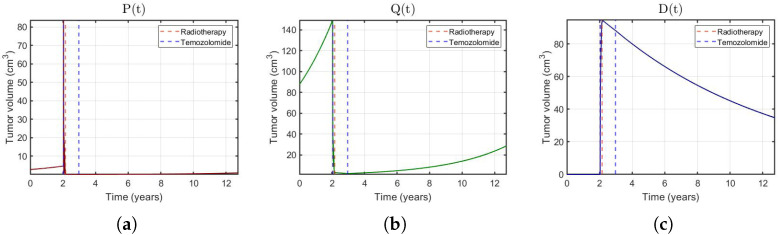
(**a**–**c**) Evolution of P(t),Q(t) and D(t) for patient 36. The sum of each of these cell populations corresponds to the total tumor volume.

Figure 2 shows some examples and, in Figure 3, the dynamics of each cell population for patient 36 is displayed. The agreement between the observed volumes and the simulated values was excellent. It can be seen that the tumor evolution was different for each patient, no matter whether RT, TMZ or both were administered. In addition, the dynamics of the virtual Ki-67 reflects the low mitotic activity of LGGs reported in clinical evidence [2]. Best fits for all patients are shown in the SI Section S2.

#### 3.1.2. Delaying Radiation Therapy Did Not Have an Effect on Overall Survival

**Figure 4:**
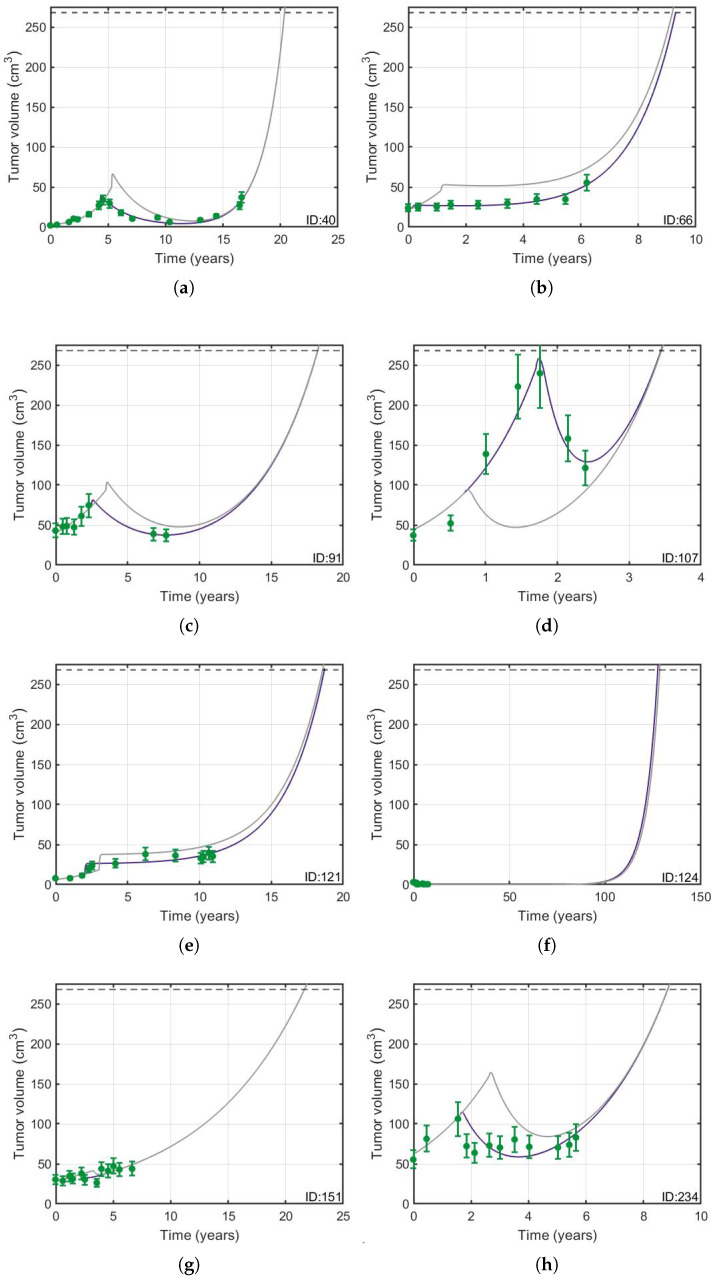
(**a**–**h**) Delaying radiotherapy does not have an effect on overall survival. RT was simulated to start one year later (gray solid line) than when actually received by the patient (solid purple line), except for patient 107, in whose case it was simulated to occur one year earlier. The horizontal black dashed line represents the fatal volume and the green circles, with their respective error bars, the longitudinal volumetric data.

The treatment starting time was moved to different times after the real time for patients who received only RT, with the exception of patient 107, in whose case it was moved to a time earlier than the real one, because at the beginning of RT the tumor volume had almost reached the fatal limit, which was taken to be 268.08 cm${}^{3}$, corresponding to the largest volume defined in Section 2.7; that is, a sphere of 8 cm in diameter. This choice allowed enough simulation time to compare the evolution of the tumor growth curves. Figure 4 shows typical results. It can be seen that the model fully agrees with the results of Van den Bent et al. [44].

### 3.2. Protracted Radiotherapy Schemes Lead to Increased Tumor Growth

**Figure 5:**
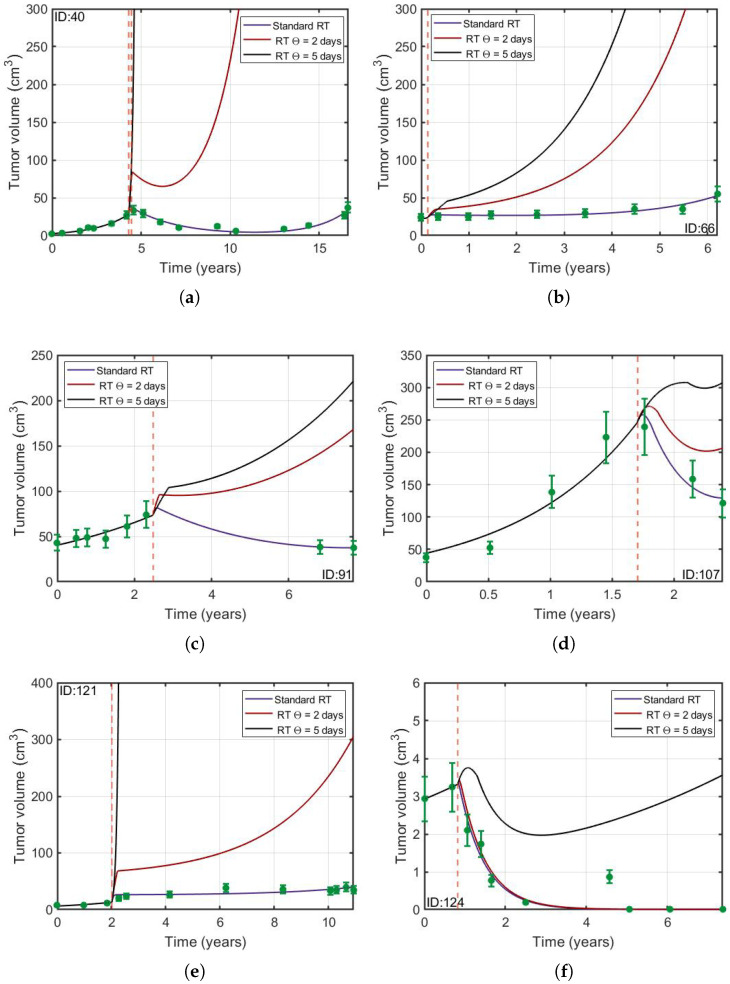

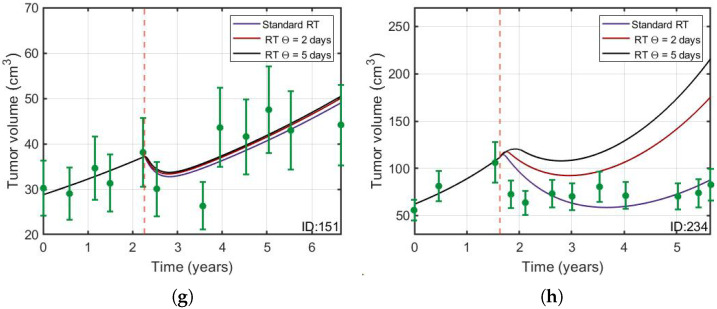
(**a**–**h**) Protracted radiotherapy schemes lead to increased tumor growth in silico. Simulations of longitudinal tumor growth dynamics of patients receiving radiation therapy fractionations spaced at 5 days (black solid line), 2 days (red solid line) and the conventional scheme (purple solid line). The vertical red dashed line shows the treatment start time and the green circles the longitudinal volumetric data.

**Figure 6:**
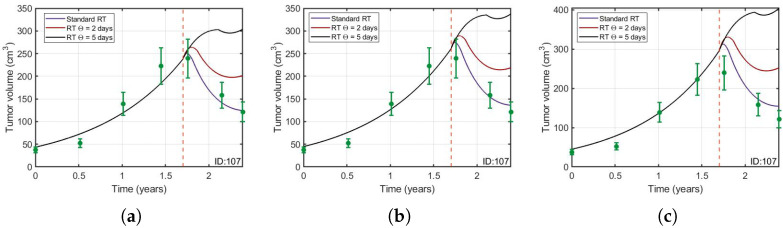
Same simulations as in Figure 5 for three different sets of parameters taking into account the 18% error in the real tumor volumes. (**a**) $V_{0}=43.56,\;\rho =0.091,\;\beta_{1}=1.08,\;\beta_{2}=0.03,\;\beta_{3}=0.0076,\;S_{q}=0.861$ and $S_{p}=0.019$. (**b**) $V_{0}=44.86,\;\rho =0.094,\;\beta_{1}=1.04,\;\beta_{2}=0.029,\beta_{3}=0.0077,\;S_{q}=0.866$ and $S_{p}=0.003$ and (**c**) $V_{0}=44.99,\;\rho =0.101,\;\beta_{1}=1,\;\beta_{2}=0.027,\;\beta_{3}=0.0081,\;S_{q}=0.869$ and $S_{p}=0$.

Some mathematical modeling work on LGGs has concluded that it is possible to space the sessions of RT without losing the therapeutic effect [18,19]. However these studies did not account for the repopulation of the compartment of proliferative cells due to the stimulation of quiescent cells after RT. We used our model to investigate the implications of considering this biological mechanism. Figure 5 displays the tumor longitudinal dynamics when the time interval between RT fractions was chosen to be either 5 days (Θ = 5 days), 2 days (Θ = 2 days) or the standard interval (Θ = 1 day with a break at weekends). In addition, we studied if these results are affected by the 18% error of the tumor volumes. Figure 6 shows that qualitatively the main results are not affected by the error. It can be seen that, at least for these specific dose interval choices, the larger the spacing between doses, the larger the final tumor volume was found to be.

### 3.3. Concurrent Cycles of RT and TMZ Produce the Longest-Lasting Therapeutic Effect

Next, we used our mathematical model as a test bed to carry out in-silico experiments, adding the missing therapy as described in Section 2.6. The parameter values of the corresponding virtual therapy are listed in Table 4. Our goal was to find optimal treatment regimens.

**Figure 7:**
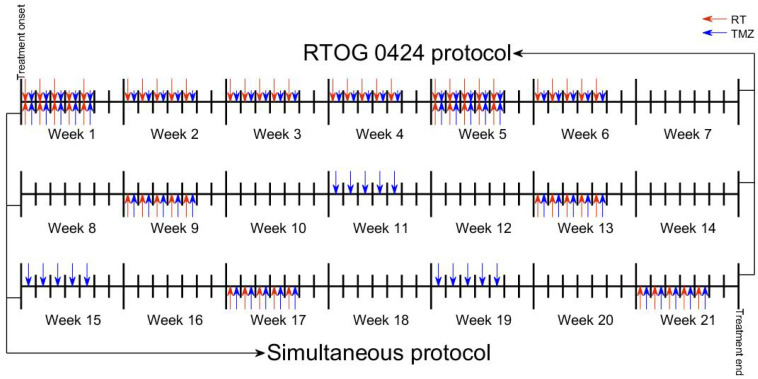
Schematic representation of the combined treatment protocols. In the RTOG 0424 protocol, RT was given concurrently with TMZ (75 mg/m${}^{2}$/day) in weeks 1–6 (top of treatment timeline) while in the simultaneous scheme, RT was only administered together with TMZ (150 mg/m${}^{2}$/day) (bottom of treatment timeline).

**Figure 8:**
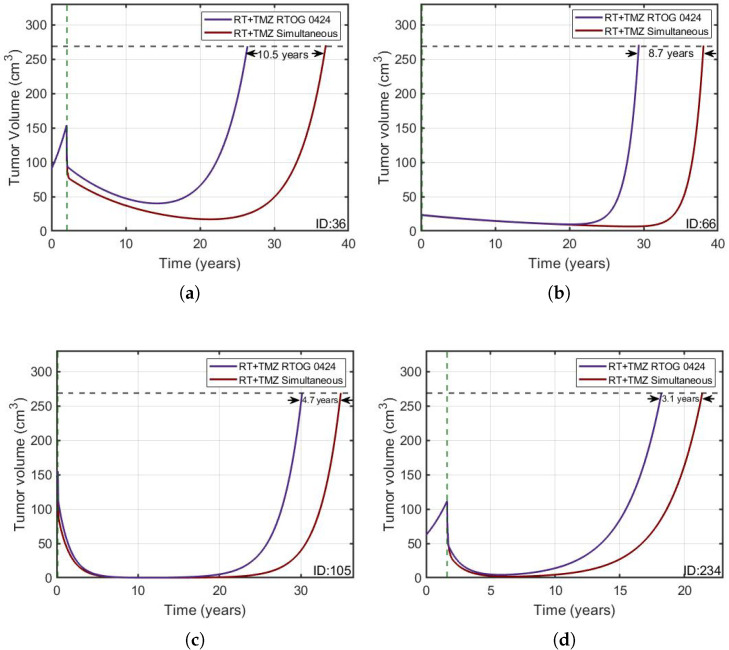
(**a**–**d**) Simulated tumor growth curves for patient 36 and for three in-silico patients treated according to RTOG 0424 (purple solid line) and the RT+TMZ simultaneous (red solid line) regimens. Treatment onset is denoted by a vertical green dashed line and the fatal tumor volume by a horizontal black dashed line.

Based on the structure of the model interactions, we hypothesized that it would be possible to space the RT sessions as long as they are administered simultaneously with TMZ, without losing therapeutic effect. The rational for this hypothesis is that radiotherapy stimulates quiescent cells (which are the largest subpopulation in LGGs) to proliferate, and once in their state of proliferation, TMZ is capable of damaging them. To test this, we computed the overall survival gain (OSG) using two different schemes. The first was concomitant radio-chemotherapy according to RTOG 0424, where RT and TMZ were started at the same time and were given concurrently in weeks 1–6. After a four-week break, TMZ cycles were given without any overlap with RT. The second scheme delivered only cycles of chemotherapy, but radiotherapy (until the course was completed) was given only on the days where chemotherapy was administered (see Figure 7). Matching both therapies led to a longer OS (Figure 8). Similar results were obtained for patient 36 and all of the virtual patients (see Table 4).

### 3.4. In-Silico Simulation of Clinical Trials

**Figure 9:**
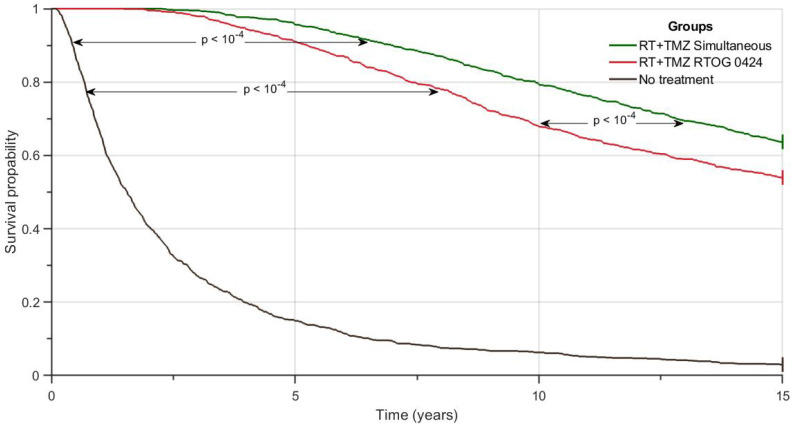
Kaplan -Meier survival curves for the control group (black line) and for the groups treated according to RTOG 0424 (red line) and the RT+TMZ simultaneous (green line) schedules. Differences between curves were statistically significant *p*-value (*p*) < 0.05.

To test the previous results, in a large enough virtual patient population, we designed an in-silico study following the methodology described in Section 2.7. A total of 3000 virtual patients were grouped into three arms. For the groups that received treatment, the numbers of TMZ cycles and RT sessions were as described in Section 2.6. Patients surviving more than 15 years in silico were considered to be censored events. Figure 9 shows the Kaplan-Meier curves for overall survival. It is clear that the simultaneous RT+TMZ protocol had the best results in terms of survival.

According to the log-rank test, the differences between all the survival curves were statistically significant (*p*-value $<10^{-4}$).

One of the most important questions in clinical trials is to estimate the number of patients required to achieve significance.

**Figure 10:**
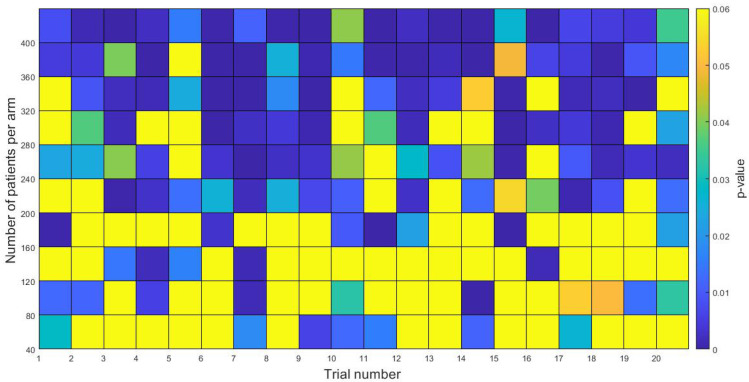
In-silico studies comparing the KM survival curves for virtual patients with a followup period of 15 years treated according to RTOG 0424 vs simultaneous RT+TMZ. The pseudocolor plot shows the *p*-values for each trial as the number of patients per arm is increased. For a patient population greater than 720 (360 per arm) the probability of finding statistically significant differences between curves is ≥95%.

To obtain that number computationally, we performed an in-silico study comparing the potential advantages of the RT+TMZ simultaneous schedule over the RTOG 0424 protocol. 80 virtual patients were equally distributed in a two-arms (RT+TMZ simultaneous and RTOG 0424 protocols) in-silico trial. The virtual follow-up period was 15 years. The p-value for the difference between the survival curves of each arm was calculated. We repeated the same experiment for another different 80 virtual patients and we followed the same process until completing 20 trials. Then the number of patients was increased by 80 (40 per arm) until a number of 800 was reached and the same methodology explained above was applied. The results are shown in Figure 10. It can be seen in Figure 10 that, as the number of patients increased, more trials were statistically significant, and in fact, for N≥360, only one trial out of 20 resulted in p>0.05. For a population of 720 patients (360 per arm) or more, the probability of finding a positive result in the trial would be very high (≥95%).

## 4. Discussion and Conclusions

In this paper, we have studied in silico what would be the most effective way, in terms of tumor control, of delivering a combination treatment of radiation therapy and temozolomide for LGGs. Radiation therapy was assumed to consist of 30 fractions of a standard 1.8 Gy dose given daily excluding weekends. Chemotherapy cycles consisted of five consecutive daily doses followed by a rest period. To find the best therapeutic combinations we used a biomathematical model. Several key biological aspects were incorporated into the mathematical model, such as the presence of a majority of quiescent cells and a small fraction of proliferative cells that were the target of cytotoxic therapies. Two different types of death process for TMZ were included, the first accounting for ‘early’ cell death and the second leading to ‘late’ cell death, as happens with RT. Finally, and as a distinctive feature of our modeling approach, we accounted for the accelerated repopulation stimulated by radiation.

It is noteworthy that the mathematical model, despite its simplicity, successfully described the tumor longitudinal volumetric dynamics of the LGG patients in our dataset treated with TMZ and/or RT. The model incorporated different parameters: two related to the initial tumor volume and composition $P_{0},Q_{0}$, four related to biological rates $\rho ,\beta_{1},\beta_{2},\beta_{3}$, two for each type of treatment, either radiotherapy $S_{q},S_{p}$ or chemotherapy $\alpha_{1},\alpha_{2}$ and one parameter related to the action of chemotherapy λ. The dynamics of the virtual Ki-67 reflected the very low mitotic activity of LGGs reported in clinical practice [2].

Many mathematical models of LGGs have been constructed to understand the response of this disease to RT [16,17,18,19,46] and chemotherapy [16,22,23,25,26]. Several previous studies [17,19] have proposed that enlarging the interval between RT fractions could be a strategy leading to survival advantages. However, with these models it is not possible to simulate the well-quantified mitotic activity of LGGs (Ki-67 labeling index around 4–5%) and also they do not consider the repopulation of proliferating cancer cells due to the stimulatory effect of RT. As seen in Figure 5, our model predicts worse tumor control when RT sessions are more spaced out. The reason is that the time interval between doses allows for quiescent stimulated cells to grow. Thus, according to our more realistic model, the non-standard schemes studied in [19] would not lead to survival advantages in their clinical application.

To our knowledge, this is the first time that the stimulatory effect produced by radiotherapy on quiescent cells has been taken into account in a mathematical model of LGGs. This effect makes quiescent cells proliferate making the chemotherapy treatment delivered concomitantly more effective. This is a key biological effect of radiotherapy that has to be taken into account in mathematical models to avoid obtaining unrealistic results, at least in the range of doses per fraction typically used for LGGs.

We found that our model is in line with the clinical trial by Van den Bent et al. [44], in the sense that deferring RT in LGGs does not alter survival time. A similar scenario was found with the administration of TMZ, where advancing or delaying treatment did not affect overall survival [26].

The main clinical contribution of our virtual simulations was that the simultaneous RT+TMZ protocol produced an overall survival gain. These in-silico gains are substantial when compared with the long-term results of the Therapy Oncology Group 0424 clinical trial carried out on High-Risk LGG patients [5]. Our research opens the door to new RT+TMZ regimens where RT fractions match TMZ treatment, allowing patients to have longer recovery times between cycles of RT/TMZ, and therefore fewer side effects than the RTOG 0424 protocol.

Our computational study predicts that a clinical trial with 700 patients would provide most likely a positive result showing the effectiveness of this therapeutic schedule. These numbers may seem large to be implemented in a real study. However, we included both virtual astrocytoma and oligodendroglioma patients as in our real cohort. Since astrocytoma patients have a shorter survival, a proof of concept study could focus only on those patients were much lower numbers would suffice.

Although the model faithfully reproduced basic aspects of radiotherapy and TMZ treatment, it has several limitations. The first is that we only had longitudinal volumetric data, thus the model was built to describe tumor evolution as a whole. Such models cannot provide information on how tumor cell density is spatially distributed throughout the brain, and do not account for details of the well-known infiltrative nature of gliomas. An extension of this work could be based on reaction-diffusion models accounting for spatial effects. The second is that, even though there are many studies on the repopulation of cancer cells during RT, this mechanism has not yet been proved in LGGs. In our modeling approach, we did not consider the development of resistance to TMZ that is known to arise after long treatments with the drug [47]. This is why we focused our study on treatments with limited number of cycles and in optimizing the outcome from combinations with RT. To develop a mathematical study of long maintenance treatments with TMZ, one should incorporate additional compartments of resistant and probably persister cells and describe the effect of the treatment in inducing transitions between them.

Since the current dose of radiotherapy is not evidence-based, it would be of the highest scientific interest to use a mathematical approach as the one presented here to find the best dose and fractionation scheme to treat LGGs. However, scarcity of longitudinal data of tumors treated with different radiation therapy schedules would limit the relevance of the results. In Ref. [19], the authors studied the problem theoretically using a linear-quadratic model of response to RT and proposed protracted schemes as the ones best controlling LGG growth. However, without the support of real-world data, it is unclear what would be the performance of those schemes when adding RT stimulatory effects and TMZ.

The main objective of this study was to provide a theoretical framework for the synergy of the simultaneous administration of both TMZ and RT in LGGs, which was not affected by the 18% error of real tumor volume as shown in the simulations. Using this model and the methodology developed here a wider range of possibilities, for example, RT+TMZ different to the protocols here studied, only RT for various doses and schedules and only TMZ in diverse regimens could be analyzed in further works. However, additional data supporting the parameter choice would be necessary.

Since it is very difficult to test the effectiveness of long-cycle strategies either in vitro or in animal models, it is a good example of how computational mathematical models can be useful in providing some preclinical evidence.

We hope this computational study will provide a theoretical grounding for the development of clinical and experimental studies and for the definition of standardized optimal treatment protocols combining TMZ and RT for low-grade glioma patients, with improved survival and better quality of life.

## Figures and Tables

**Table 1:** Patients included in the study. Patient 36 was the only one treated with concurrent RT and TMZ. Patient 151 received RT and 11 years later TMZ. The meaning of the abbreviations used in the table can be found at the end of this document.

ID	Age at Diagnosis	Sex	Histology	Treatment	Ki-67 Labeling Index (%)	#RT Sessions	#TMZ Cycles
10	48	F	O	TMZ	5	-	15
25	47	M	O	TMZ	5	-	4
36	40	M	O	TMZ & RT	3	35	13
40	30	F	A	RT	1	34	-
57	54	M	O	TMZ	4	-	10
66	28	M	A	RT	NA	30	-
91	34	M	A	RT	NA	30	-
105	33	M	O	TMZ	3	-	20
107	34	M	A	RT	3	30	-
108	46	M	O	TMZ	3	-	5
121	48	M	A	RT	3	35	-
124	27	M	OA	RT	3	35	-
151	58	M	O	RT	3	7	-
				TMZ	5	-	13
159	46	M	NA	TMZ	NA	-	12
203	51	M	O	TMZ	6	-	10
213	58	F	O	TMZ	3	-	20
234	44	M	A	RT	2	30	-

**Table 2:** Parameters describing TMZ concentration in brain tumor tissue.

Parameter	Description	Value	Sources
*c*	Standard dose per day	150 mg/m${}^{2}$	[26]
*b*	Patient body surface	1.9 m${}^{2}$ (men)	[41]
		1.6 m${}^{2}$ (women)	[41]
γ	Fraction of TMZ reaching	2.1·$10^{-6}/$mL (men)	[25]
	the brain interstitium	$2.5\;\cdot \;10^{-6}/$mL (women)	[42]
$t_{1/2}$	TMZ half-life clearance time	≃2 h	[43]
λ	Rate of TMZ decay	0.3466/h	[25]

**Table 3:** Parameter values obtained for the best fits of patients to Equation (1). Patient 151 was considered independently for each therapy. The units for the initial conditions and parameters are: for $P_{0}$ and $Q_{0}$, cm${}^{3}$; for Ki-67${}_{0}$→%; for ρ, $\beta_{1}$, $\beta_{2}$ and $\beta_{3}$, 1/day. Finally, $\alpha_{1}$ and $\alpha_{2}$ are measured in cm${}^{3}$/(μg day). * Estimated values.

ID	#RT Sessions	#TMZ Cycles	$P_{0}$	$Q_{0}$	Ki-67${}_{0}$	ρ	$\beta_{1}$	$\beta_{2}$	$\beta_{3}$	$S_{q}$	$S_{p}$	$\alpha_{1}$	$\alpha_{2}$
**Radiotherapy + Temozolomide**													
36	35	13	2.7	87.6	3	0.023	1.5	0.046	0.0002	0.45	0.55	2.3	3.9
**Radiotherapy**													
40	34	-	0.02	2.5	1	0.15	1	0.008	0.0011	0.32	0	-	-
66	30	-	0.64	20.9	3 *	0.06	1.5	0.04	0.0001	0.39	0	-	-
91	30	-	0.8	39.5	2 *	0.033	2.1	0.04	0.0008	0.16	0.04	-	-
107	30	-	1.3	42.8	3	0.092	1.08	0.03	0.0078	0.86	0	-	-
121	35	-	0.18	6	3	0.1	0.33	0.002	$1\times 10^{-5}$	0.1	0.7	-	-
124	35	-	0.08	2.8	3	0.013	0.87	0.02	0.004	0.01	0	-	-
151	7	-	0.86	27.9	3	0.01	1.3	0.04	0.01	0.94	0.09	-	-
234	30	-	1.2	6.9	2	0.032	1.6	0.04	0.0024	0.78	0	-	-
**Temozolomide**													
10	-	15	2.2	43.6	5	0.01	3.5	0.184	0.0008	-	-	6.01	4.31
25	-	4	1.7	32.6	5	0.028	6.42	0.33	0.004	-	-	20	20
57	-	10	1.8	45.1	4	0.01	0.23	0.009	0.006	-	-	15	9.8
105	-	20	4.3	141.7	3	0.035	0.7	0.02	0.0018	-	-	2.8	6.5
108	-	5	0.4	15.2	3	0.04	10.6	0.32	0.003	-	-	45	3
151	-	13	1.7	33.5	5	0.012	0.53	0.02	0.0013	-	-	14.8	1
159	-	12	2	66.1	3 *	0.012	3.5	0.1	0.004	-	-	11	6
203	-	10	1.7	27.2	6	0.059	1.6	0.098	0.021	-	-	8.2	0.37
213	-	20	0.7	23.1	3	0.011	4.7	0.14	0.0005	-	-	15.2	15.1

**Table 4:** Real (in black, see Table 3) and virtual (in blue) values of the parameters related to therapies and the numbers of TMZ cycles and RT sessions received for the patients included. $\alpha_{1}$ and $\alpha_{2}$ are measured in cm${}^{3}$/(μg day). The final column corresponds to the overall survival gain (OSG) for each patient.

ID	#RT Sessions	#TMZ Cycles	$S_{q}$	$S_{p}$	$\alpha_{1}$	$\alpha_{2}$	OSG (Years)
10	30	15	0.28	0.47	6.01	4.31	>10
25	30	4	0.1	0.65	20	20	−1.2
36	35	13	0.45	0.55	2.3	3.9	>10
40	34	12	0.32	0	34.6	14.9	>10
57	30	10	0.68	0.69	15	9.8	8
66	30	12	0.39	0	30.3	1	8.7
91	30	12	0.16	0.04	7.7	18.3	>10
105	30	20	0.51	0	2.8	6.5	4.7
107	30	12	0.86	0	30.3	3.7	0.4
108	30	5	0.2	0.48	45	3	2.9
121	35	12	0.1	0.7	10.3	10	>10
124	35	12	0.01	0	14.1	13.7	>10
151	7	12	0.94	0.09	5.5	1.4	0.26
151	30	13	0.83	0.24	14.8	1	1.9
159	30	12	0.18	0.17	11	6	>10
203	30	10	0.6	0.27	8.2	0.37	1.4
213	30	20	0.36	0.41	15.2	15.1	>10
234	30	12	0.78	0	2.5	15.6	3.1

## Data Availability

The study was approved by Kantonale Ethikkommission Bern (Bern, Switzerland), with approval number: 07.09.72.

## Supplementary Materials

The following are available online at https://www.mdpi.com/2075-4426/11/10/1036/s1.

## Abbreviations

The following abbreviations are used in this manuscript:

LGG	Low-Grade Glioma
O	Oligodendroglioma
A	Astrocytoma
OA	Oligoastrocytoma
RT	Radiation Therapy
TMZ	Temozolomide
CS	Cycle Size
PCV	Procarbazine, Lomustine and Vincristine
MRI	Magnetic Resonance Imaging
OSG	Overall Survival Gain
NA	Not Available
