# Supplementary material for: Optimal Combinations of Chemotherapy and Radiotherapy in Low-Grade Gliomas: A Mathematical Approach
Source: J Pers Med. 2021 Oct 16;11(10):1036. doi: 10.3390/jpm11101036 (PMC8537400; doi:10.3390/jpm11101036)
Supplement: Supplementary file 1 [file jpm-11-01036-s001.zip › jpm-1366364-SI/SIv2.pdf]

Supplementary Material for
Optimal combinations of chemotherapy and radiotherapy for low-grade gliomas: A mathematical approach

Luis E. Ayala-Hernández, Armando Gallegos, Philippe Schutz, Michael Murek, Luis Pï¿½rez-Romasanta, Juan Belmonte-Beitia and Víctor M. Pérez-García

1 Exact solutions for Eqs. (2)

In this supplementary material, we solve the following system of linear ordinary differential equations:

$$\begin{aligned} \frac{dP}{dt} &= \rho P - \beta_1 P + \beta_2 Q \\ \frac{dQ}{dt} &= \beta_1 P - \beta_2 Q \end{aligned} \tag{S1}$$

System (S1) can be written as

$$\begin{pmatrix} \dot{P} \\ \dot{Q} \end{pmatrix} = \begin{pmatrix} \rho - \beta_1 & \beta_2 \\ -\beta_1 & \beta_2 \end{pmatrix} \begin{pmatrix} P \\ Q \end{pmatrix}$$

whose eigenvalues and the corresponding eigenvectors are,

$$\begin{aligned} \lambda_1 &= \frac{\rho - \beta_1 - \beta_2 + \sqrt{(\rho - \beta_1 - \beta_2)^2 + 4\beta_1\beta_2}}{2}, \qquad \vec{v}_1 = \begin{pmatrix} \frac{\beta_2 + \lambda_1}{\beta_1} \\ 1 \end{pmatrix} \\ \lambda_2 &= \frac{\rho - \beta_1 - \beta_2 - \sqrt{(\rho - \beta_1 - \beta_2)^2 + 4\beta_1\beta_2}}{2}, \qquad \vec{v}_2 = \begin{pmatrix} \frac{\beta_2 + \lambda_2}{\beta_1} \\ 1 \end{pmatrix} \end{aligned}$$

Thus, the solution of the system is given by

$$\begin{pmatrix} P \\ Q \end{pmatrix} = C_1 \begin{pmatrix} \frac{\beta_2 + \lambda_1}{\beta_1} \\ 1 \end{pmatrix} e^{\lambda_1 t} + C_2 \begin{pmatrix} \frac{\beta_2 + \lambda_2}{\beta_1} \\ 1 \end{pmatrix} e^{\lambda_2 t}$$

Using the initial conditions of the problem for $t_0 = 0$, i.e. $P(0) = P_0$ and $Q(0) = Q_0$, and after some algebra, we obtain the solution of the system of differential equations

$$\begin{aligned} P(t) &= \frac{\beta_2 + \lambda_1}{\beta_1} \left(\frac{\beta_2 + \lambda_2}{\lambda_2 - \lambda_1} Q_0 - \frac{\beta_1}{\lambda_2 - \lambda_1} P_0 \right) e^{\lambda_1 t} + \frac{\beta_2 + \lambda_2}{\beta_1} \left(\frac{\beta_1}{\lambda_2 - \lambda_1} P_0 - \frac{\beta_2 + \lambda_1}{\lambda_2 - \lambda_1} Q_0 \right) e^{\lambda_2 t} \\ Q(t) &= \left(\frac{\beta_2 + \lambda_2}{\lambda_2 - \lambda_1} Q_0 - \frac{\beta_1}{\lambda_2 - \lambda_1} P_0 \right) e^{\lambda_1 t} + \left(\frac{\beta_1}{\lambda_2 - \lambda_1} P_0 - \frac{\beta_2 + \lambda_1}{\lambda_2 - \lambda_1} Q_0 \right) e^{\lambda_2 t} \end{aligned}$$

2 Fitting of model Equations (1) for all patients included in the study

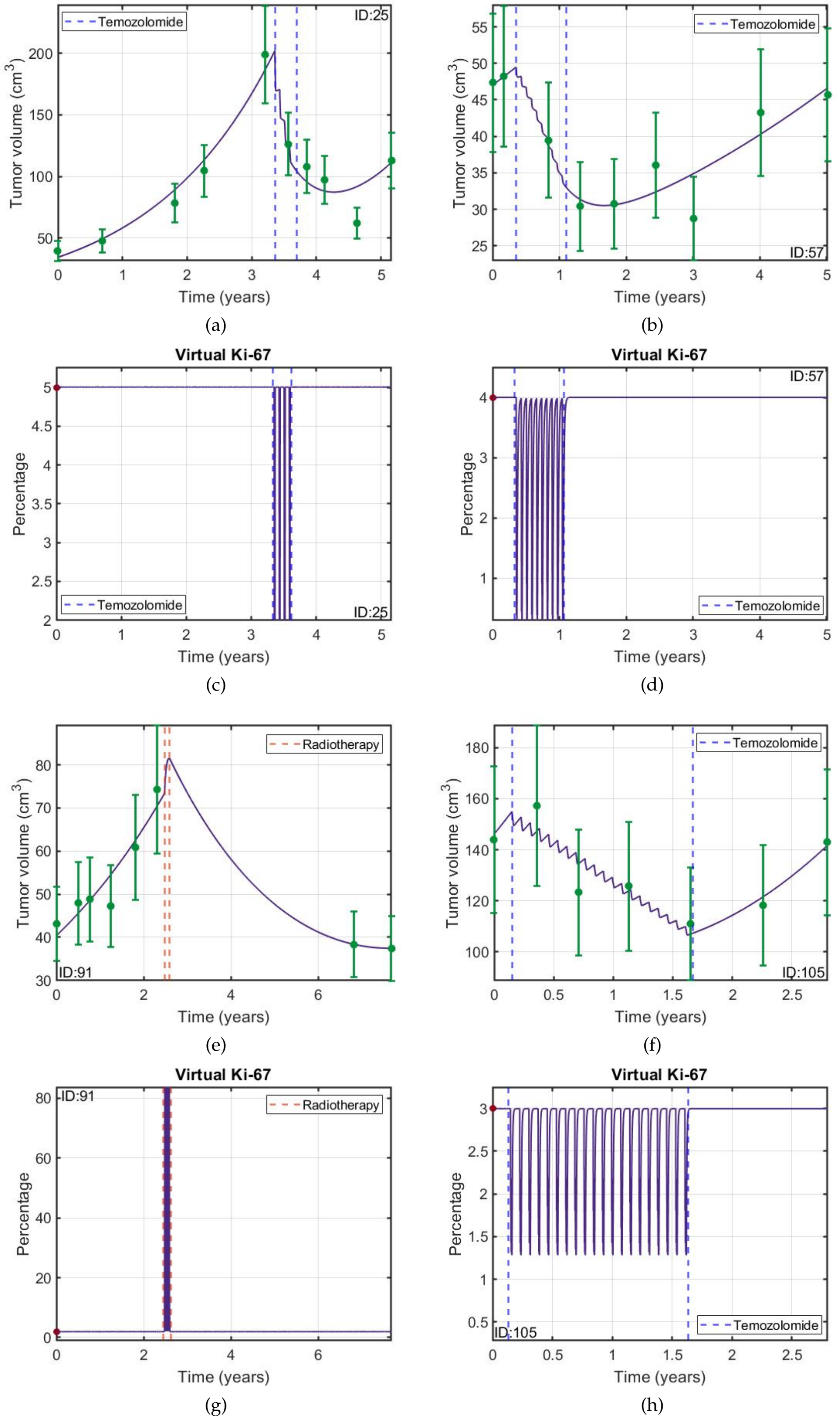

Figure S1: (a,b,e,f) Longitudinal volumetric dynamics (green circles) and best fits obtained using Eqs. (1) (solid purple line). Data shown correspond to patients 25, 57, 91 and 105. (c,d,g,h) Time evolution of $100P(t)/(P(t) + Q(t))$ interpreted as virtual (solid purple line) and initial (red circle) Ki-67 labeling indexes. The vertical dashed lines indicates the onset and end times of the treatments received by each patient (either RT or QT).

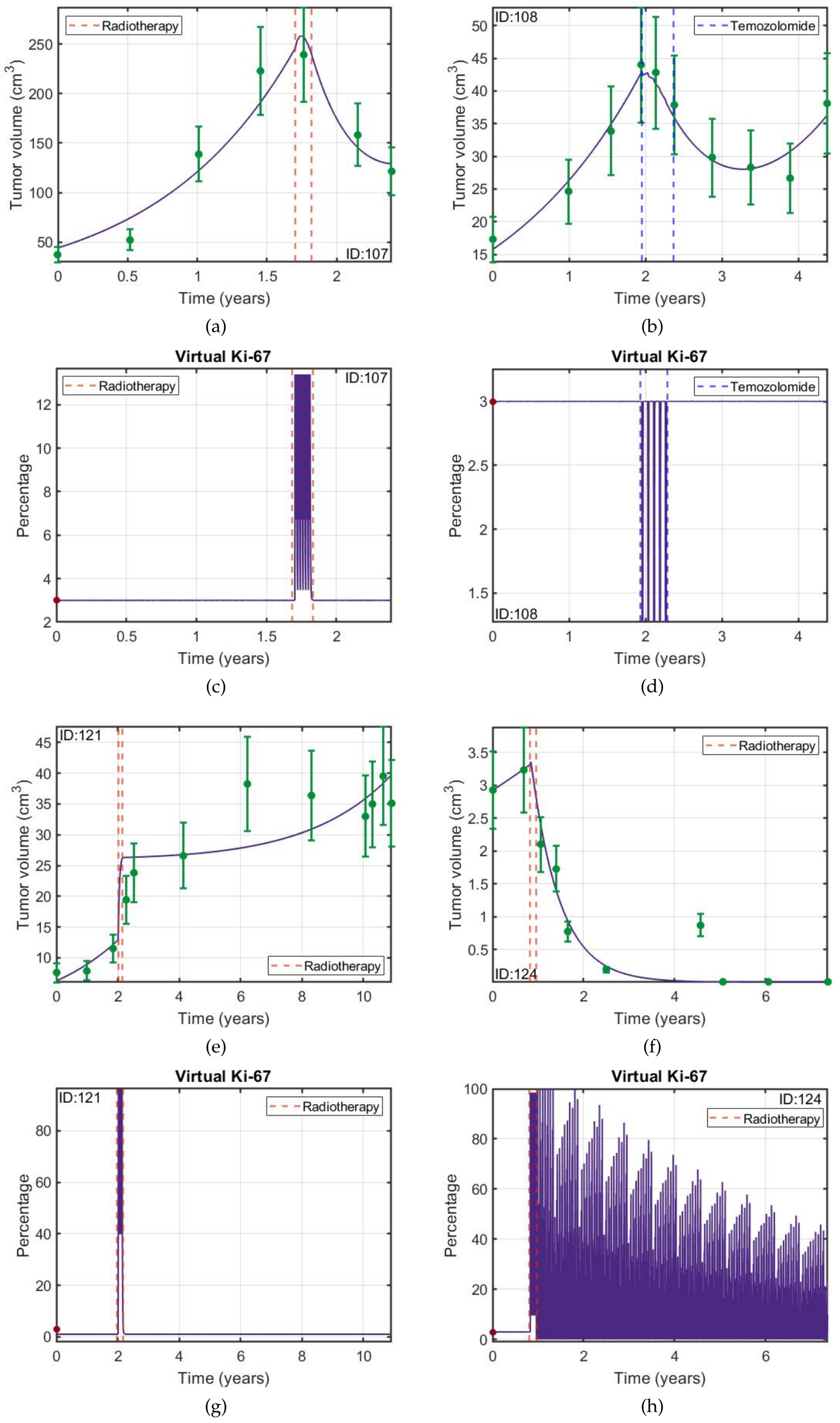

Figure S2: (a,b,e,f)Longitudinal volumetric dynamics (green circles) and best fits obtained using Eqs. (1) (solid purple line). Data shown correspond to patients 107, 108, 121 and 124 (green circles). (c,d,g,h) Time evolution of $100P(t)/(P(t)+Q(t))$ interpreted as virtual (solid purple line) and initial (red circle) Ki-67 labeling indexes. The vertical dashed lines indicates the onset and end times of the treatments received by each patient (either RT or QT). The behaviour in (h) is due to the tumor volume going to zero after treatment.

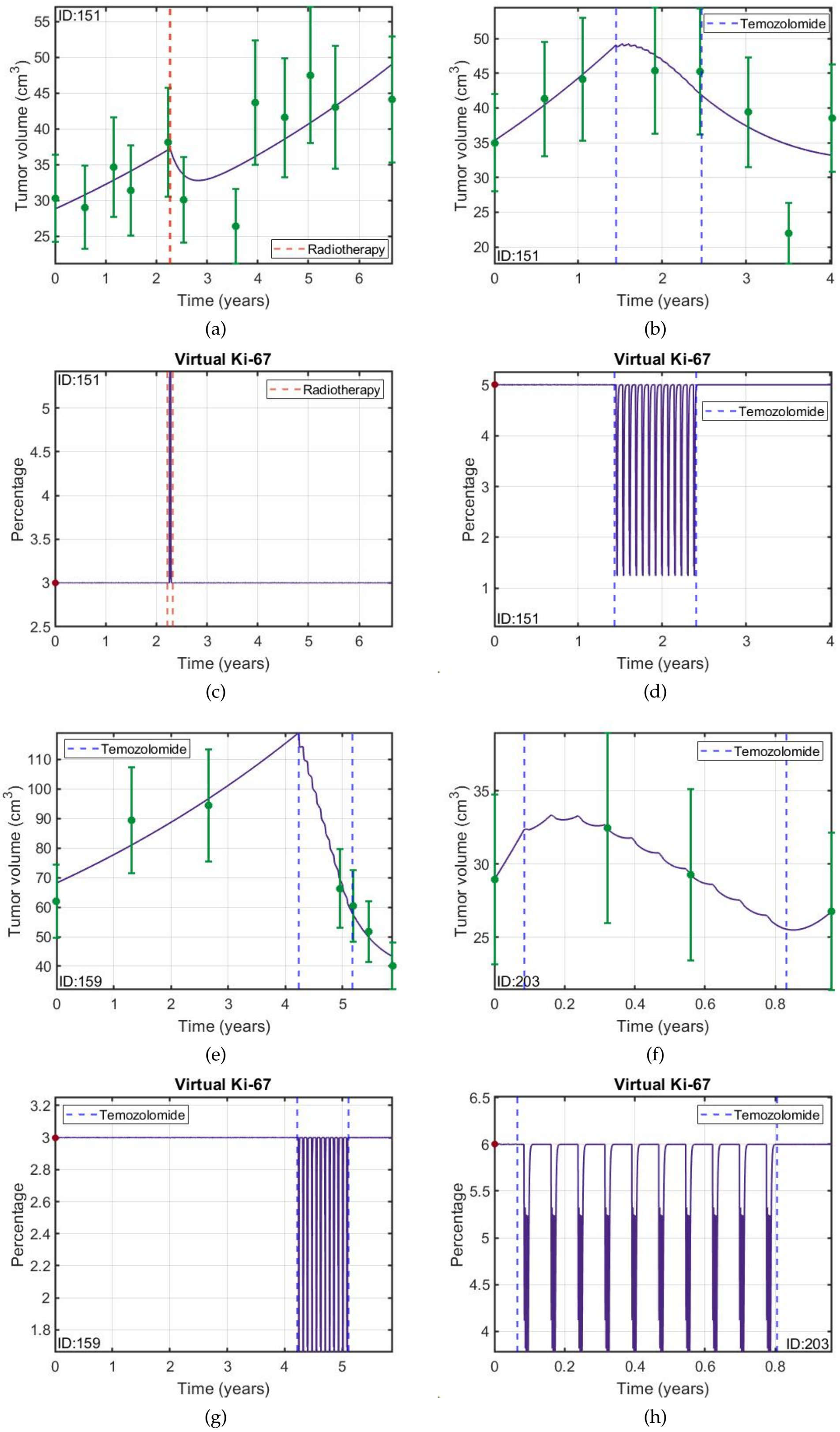

Figure S3: (a,b,e,f) Longitudinal volumetric dynamics (green circles) and best fits obtained using Eqs. (1) (solid purple line). Data shown correspond to patients 151, 159 and 203 (green circles). (c,d,g,h) Time evolution of $100P(t)/(P(t)+Q(t))$ interpreted as virtual (solid purple line) and initial (red circle) Ki-67 labeling indexes. The vertical dashed lines indicates the onset and end times of the treatments received by each patient (either RT or QT).

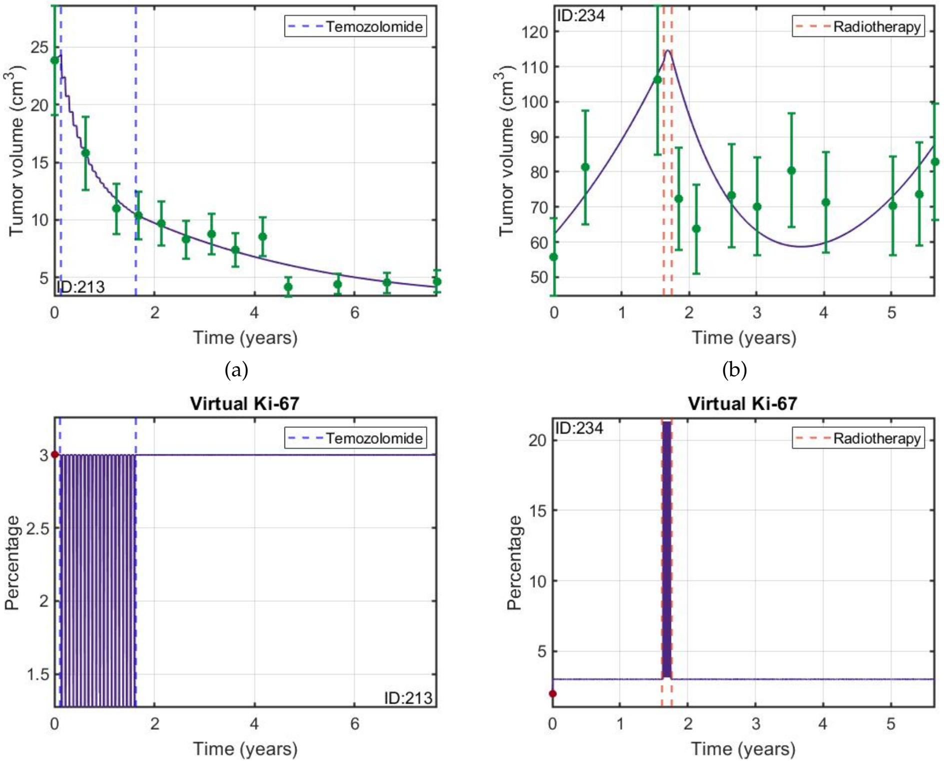

(a) (b) (c) (d)

Figure S4: (a,b) Longitudinal volumetric dynamics (green circles) and best fits obtained using Eqs. (1) (solid purple line). Shown are data for patients 213 and 234 (green circles). (c,d) Time evolution of $100P(t)/(P(t)+Q(t))$ interpreted as virtual (solid purple line) and initial (red circle) Ki-67 labeling indexes. The vertical dashed lines indicates the onset and end times of the treatments received by each patient (either RT or QT).
