# Supplementary figures and images for: Optimal Combinations of Chemotherapy and Radiotherapy in Low-Grade Gliomas: A Mathematical Approach
Source: J Pers Med. 2021 Oct 16;11(10):1036. doi: 10.3390/jpm11101036 (PMC8537400; doi:10.3390/jpm11101036)

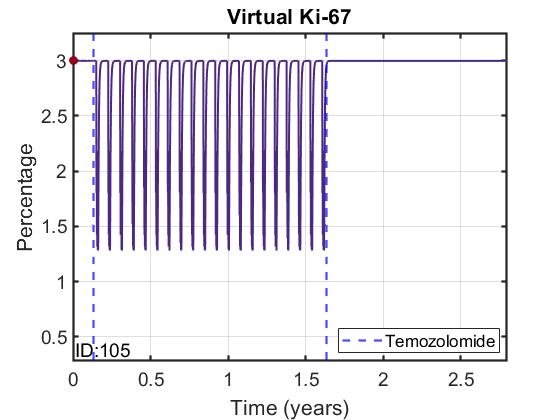

Supplement: Supplementary file 1 [file jpm-11-01036-s001.zip › jpm-1366364-SI/P105ki.jpg]

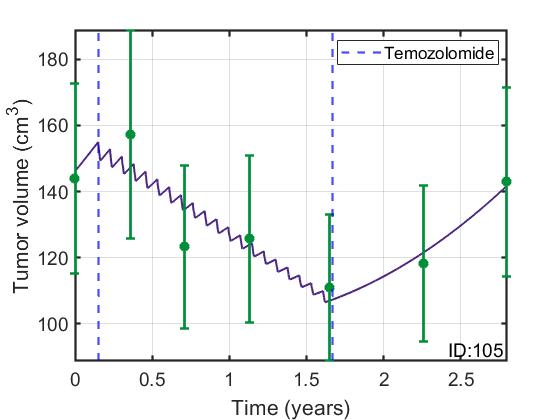

Supplement: Supplementary file 1 [file jpm-11-01036-s001.zip › jpm-1366364-SI/P105v.jpg]

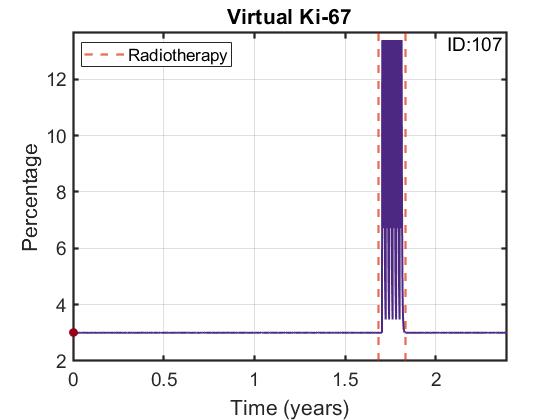

Supplement: Supplementary file 1 [file jpm-11-01036-s001.zip › jpm-1366364-SI/P107ki.jpg]

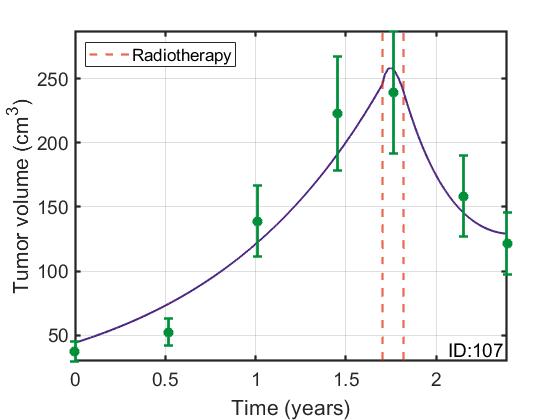

Supplement: Supplementary file 1 [file jpm-11-01036-s001.zip › jpm-1366364-SI/P107v.jpg]

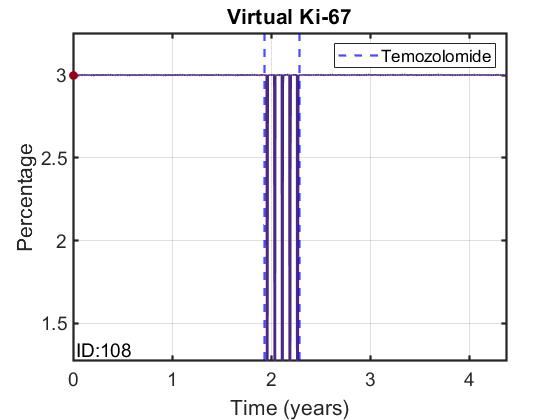

Supplement: Supplementary file 1 [file jpm-11-01036-s001.zip › jpm-1366364-SI/P108ki.jpg]

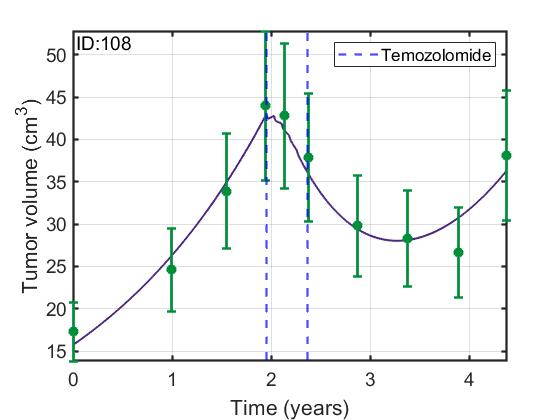

Supplement: Supplementary file 1 [file jpm-11-01036-s001.zip › jpm-1366364-SI/P108v.jpg]

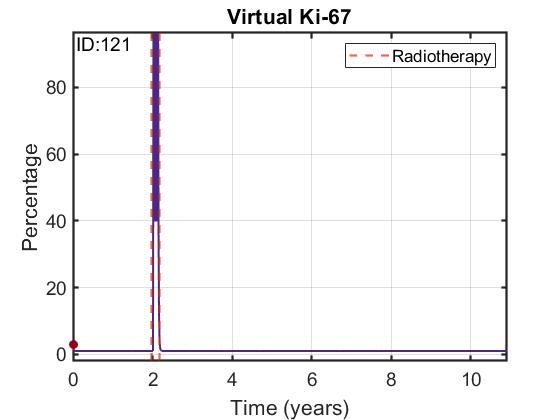

Supplement: Supplementary file 1 [file jpm-11-01036-s001.zip › jpm-1366364-SI/P121ki.jpg]

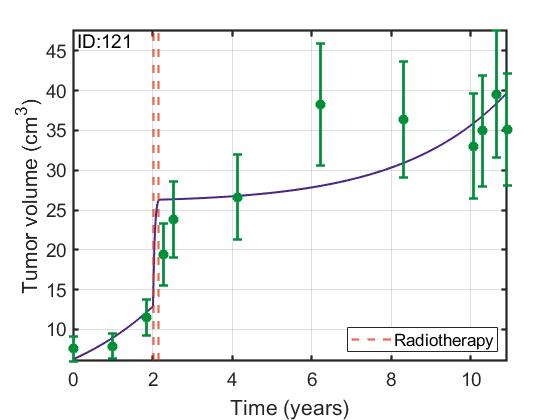

Supplement: Supplementary file 1 [file jpm-11-01036-s001.zip › jpm-1366364-SI/P121v.jpg]

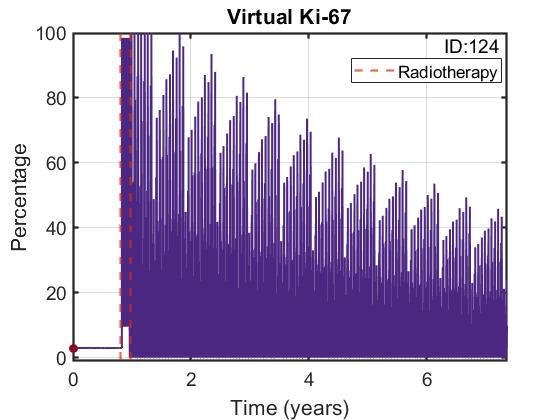

Supplement: Supplementary file 1 [file jpm-11-01036-s001.zip › jpm-1366364-SI/P124ki.jpg]

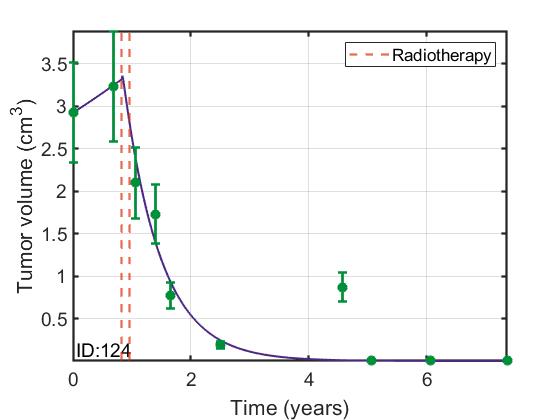

Supplement: Supplementary file 1 [file jpm-11-01036-s001.zip › jpm-1366364-SI/P124v.jpg]

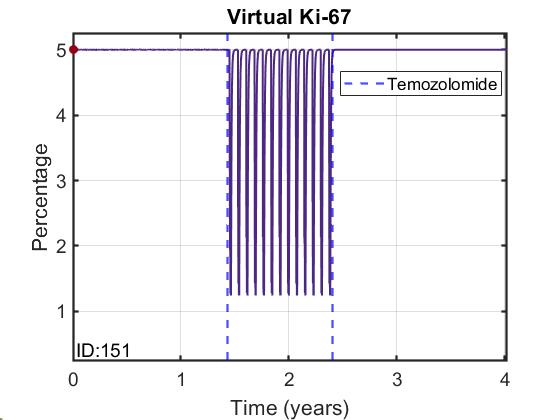

Supplement: Supplementary file 1 [file jpm-11-01036-s001.zip › jpm-1366364-SI/P151Qki.jpg]

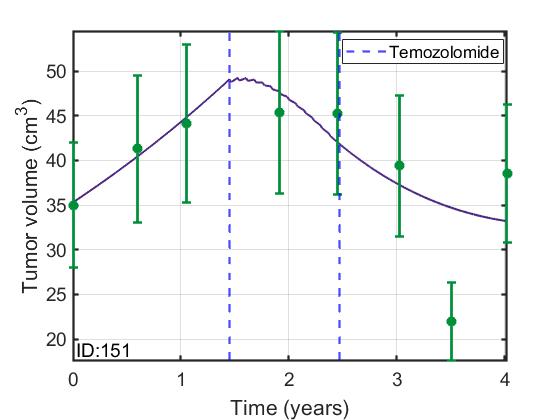

Supplement: Supplementary file 1 [file jpm-11-01036-s001.zip › jpm-1366364-SI/P151Qv.jpg]

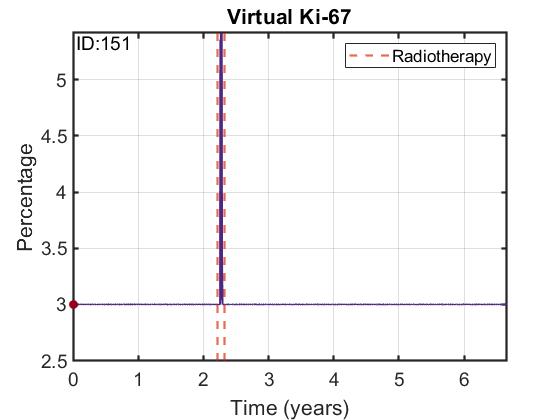

Supplement: Supplementary file 1 [file jpm-11-01036-s001.zip › jpm-1366364-SI/P151Rki.jpg]

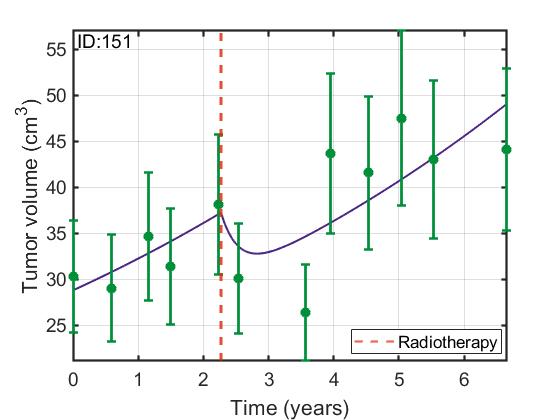

Supplement: Supplementary file 1 [file jpm-11-01036-s001.zip › jpm-1366364-SI/P151Rv.jpg]

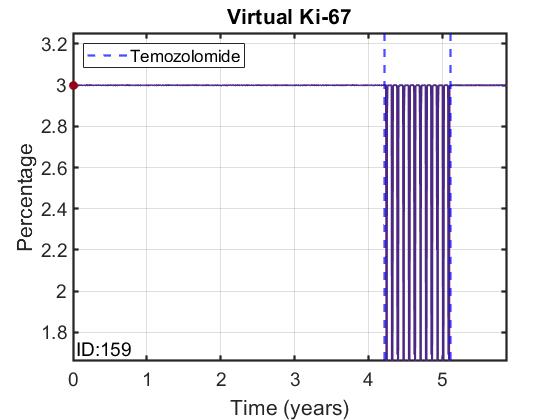

Supplement: Supplementary file 1 [file jpm-11-01036-s001.zip › jpm-1366364-SI/P159ki.jpg]

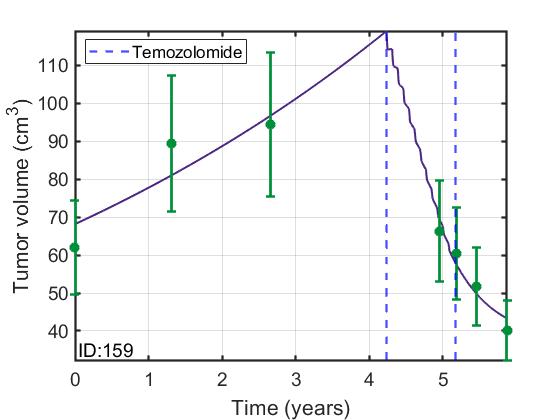

Supplement: Supplementary file 1 [file jpm-11-01036-s001.zip › jpm-1366364-SI/P159v.jpg]

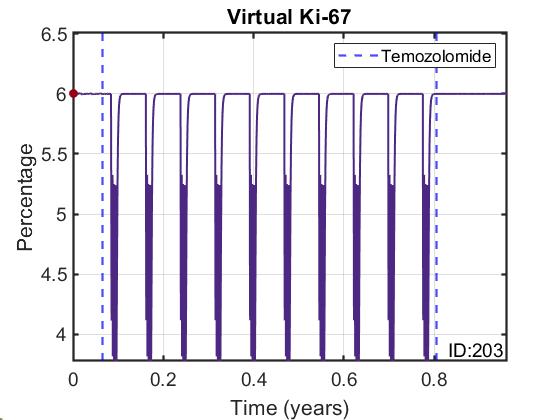

Supplement: Supplementary file 1 [file jpm-11-01036-s001.zip › jpm-1366364-SI/P203ki.jpg]

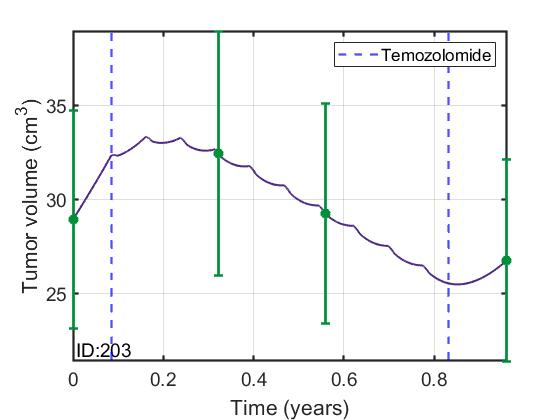

Supplement: Supplementary file 1 [file jpm-11-01036-s001.zip › jpm-1366364-SI/P203v.jpg]

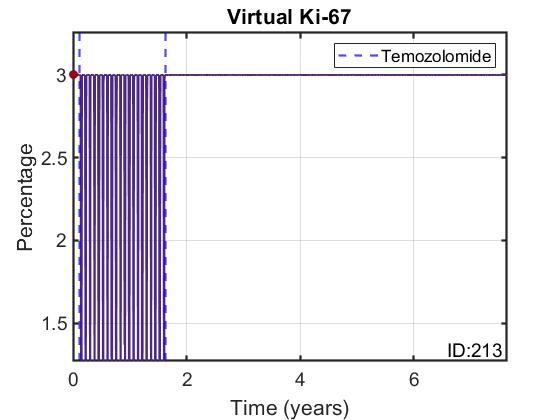

Supplement: Supplementary file 1 [file jpm-11-01036-s001.zip › jpm-1366364-SI/P213ki.jpg]

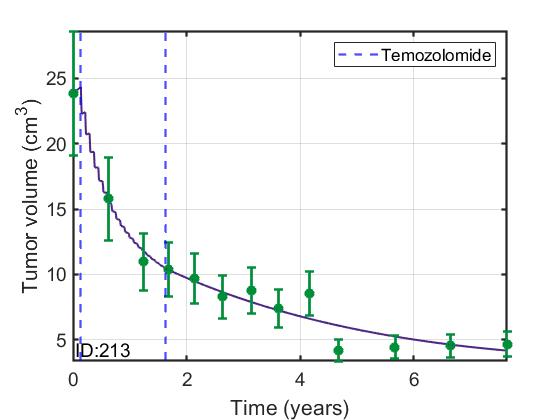

Supplement: Supplementary file 1 [file jpm-11-01036-s001.zip › jpm-1366364-SI/P213v.jpg]

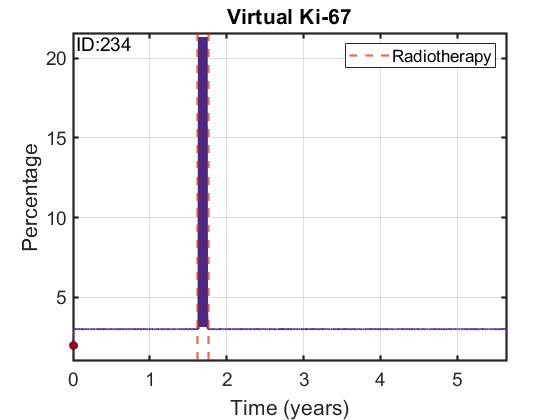

Supplement: Supplementary file 1 [file jpm-11-01036-s001.zip › jpm-1366364-SI/P234ki.jpg]

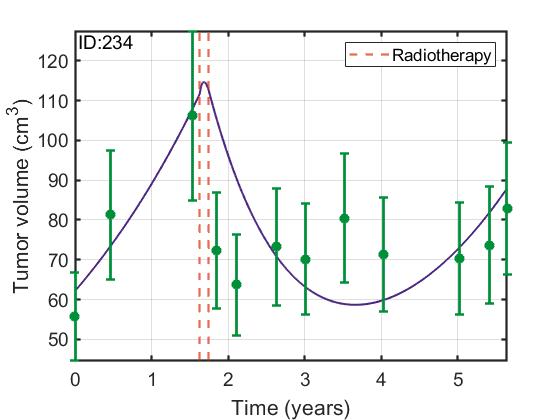

Supplement: Supplementary file 1 [file jpm-11-01036-s001.zip › jpm-1366364-SI/P234v.jpg]

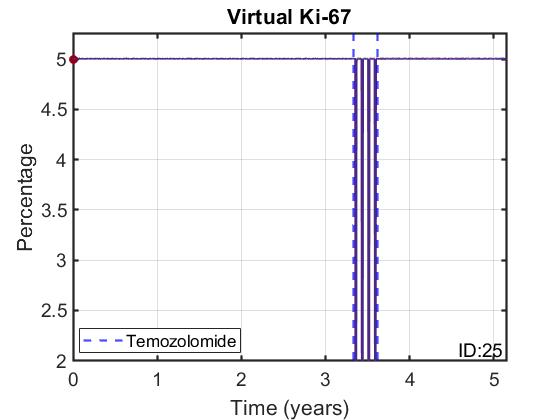

Supplement: Supplementary file 1 [file jpm-11-01036-s001.zip › jpm-1366364-SI/P25ki.jpg]

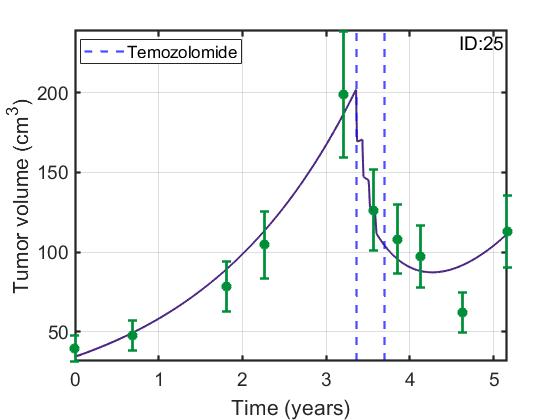

Supplement: Supplementary file 1 [file jpm-11-01036-s001.zip › jpm-1366364-SI/P25v.jpg]

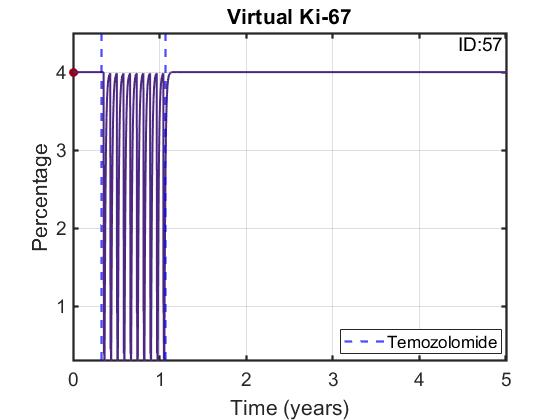

Supplement: Supplementary file 1 [file jpm-11-01036-s001.zip › jpm-1366364-SI/P57ki.jpg]

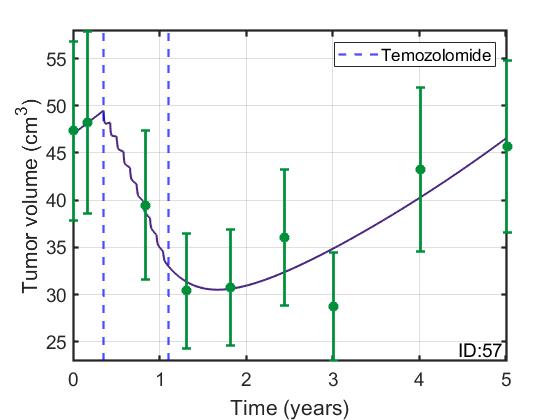

Supplement: Supplementary file 1 [file jpm-11-01036-s001.zip › jpm-1366364-SI/P57v.jpg]

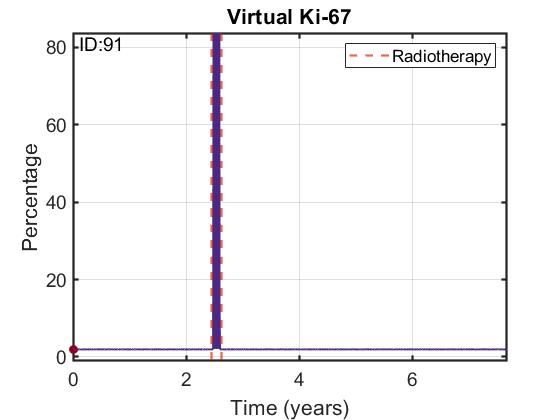

Supplement: Supplementary file 1 [file jpm-11-01036-s001.zip › jpm-1366364-SI/P91ki.jpg]

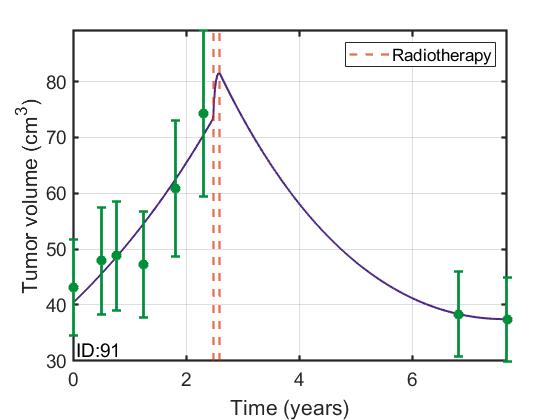

Supplement: Supplementary file 1 [file jpm-11-01036-s001.zip › jpm-1366364-SI/P91v.jpg]
